# Correction

**DOI:** 10.1111/cas.15588

**Published:** 2022-11-03

In an article^1^ titled “MMP13‐containing exosomes promote nasopharyngeal carcinoma metastasis” by Yiwen You, Ying Shan, Jing Chen, Huijun Yue, Bo You, Si Shi, Xingyu Li, Xiaolei Cao, the following errors were published:

In Figure 4, it was accidentally applied the same micrograph in Figures 4a,j. The corrected figure is shown below:

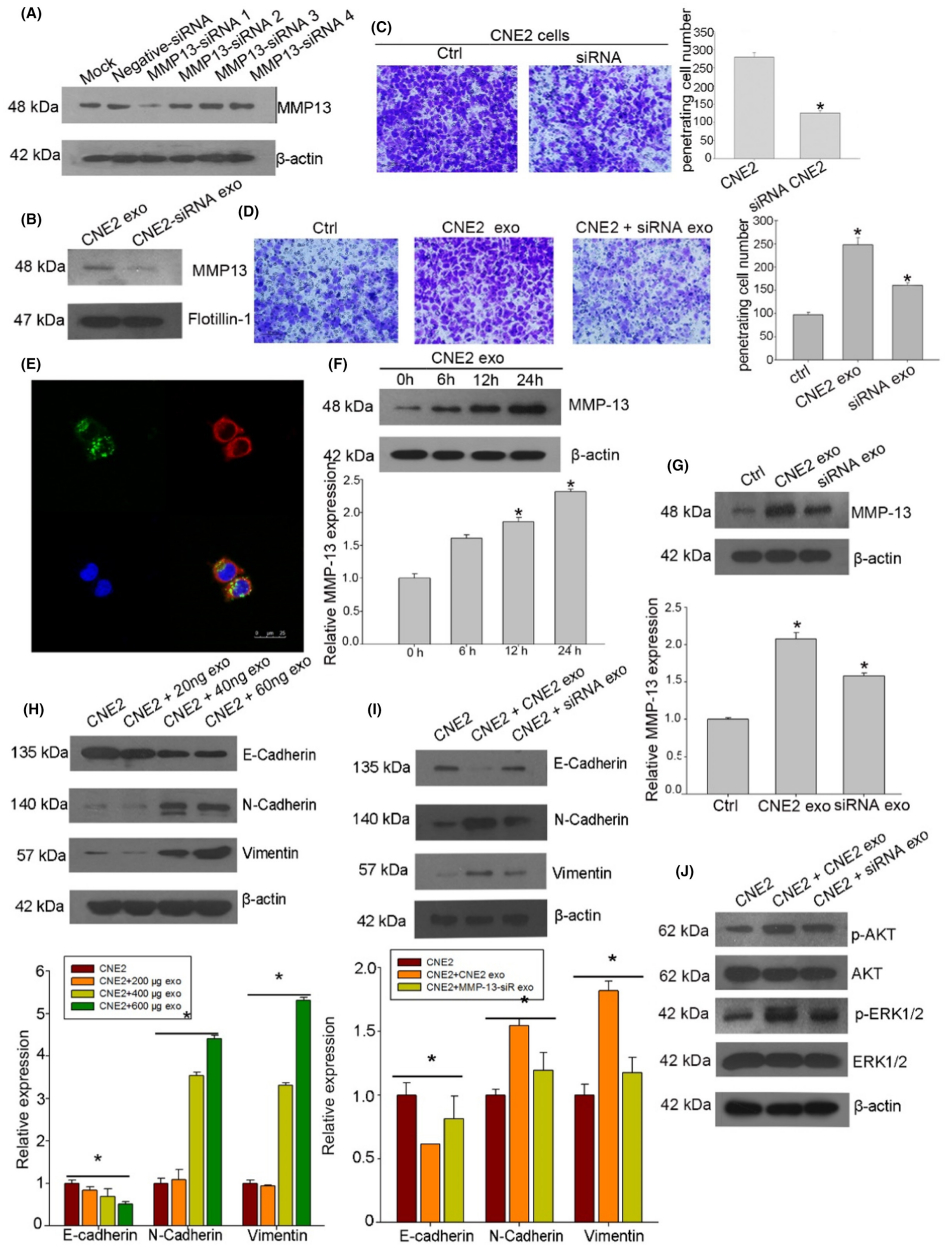

The authors apologize for the error.
